# Study of the Antioxidant Property Variation of Cornelian Cherry Fruits during Storage Using HPTLC and Spectrophotometric Assays

**DOI:** 10.1155/2016/2345375

**Published:** 2016-11-10

**Authors:** Anamaria Hosu, Claudia Cimpoiu, Luminita David, Bianca Moldovan

**Affiliations:** Faculty of Chemistry and Chemical Engineering, “Babeş-Bolyai” University, 11 Arany Janos Street, 400028 Cluj-Napoca, Romania

## Abstract

Cornus species fruits are well known as a rich source of antioxidant compounds responsible for their diverse health benefits. The present study aims to investigate the variation of the total antioxidant capacity of Cornelian cherry (*Cornus mas* L.) fruits during storage, using high-performance thin-layer chromatography (HPTLC) and two spectrophotometric assays based on different mechanisms: the 2,2-azinobis(3-ethylbenzothiazolyne-6-sulphonic acid) radical cation (ABTS^+∙^) assay and the ferric reducing antioxidant power (FRAP) assay. The fruit extract was stored at room temperature (22°C) for 19 days. No major differences in the total antioxidant capacity were observed during this period, indicating that storage does not have any deleterious effect on the antioxidant properties of the investigated fruits extract. The antioxidant capacity varied between 12.91 and 12.83 *µ*mol Trolox/g fruit as determined by the HPTLC method and from 36.13 to 33.93 *µ*mol Trolox/g fruit as determined by the ABTS assay.

## 1. Introduction

It is well known that diets rich in fruits and vegetables present numerous health benefits being particular important in prevention of some degenerative diseases, such as cancer and cardiovascular and neurological disorders [1–4]. All these biological activities can be associated with the antioxidant capacity of nutritive and nonnutritive compounds which can be found in plants and play an important role in protection against cellular oxidation processes by reacting with free radicals [5]. The best way to provide bioactive compounds with antioxidant properties to the human body is consuming large amounts of fruits rich in antioxidants. Lately, Cornelian cherries have been proved to be fruits containing high levels of secondary plant metabolites with potent antioxidant capacity [6, 7].

Although the content of single specific antioxidant compounds from fruits is an important parameter in fruits characterization, the overall nutritional value is better expressed by analyzing the total antioxidant capacity [8]. The total antioxidant capacity of fruits cannot be accurately evaluated by a single method as the different antioxidant compounds may act through different mechanisms, depending on the reaction involved and on the reaction system [9]. Therefore, elaborating an antioxidant profile of particular food stuffs requires multiple assays.

Cornelian cherries are edible, sweet-sour in taste fruits of the* Cornus mas* plant, from the dogwood family, being popular in Southern Europe and Southwest Asia.

Cornelian cherry fruits are valued as a rich source of vitamin C, sugars, organic acids, tannins, and phenolics [10]. Usually, Cornelian cherries are consumed fresh, dried, or pickled. Other processed forms, such as juice, alcoholic drinks, gels, or jams, are also used in human diet. These fruits have been traditionally used in the folk medicine and recent researches confirmed their curative properties which are due to their anti-inflammatory, antidiarrhea, antiallergic, antimicrobial, and antimalarial actions [11, 12]. All these health benefits increased the interest in these fruits, studies being recently conducted to investigate their physical and chemical properties and to elucidate their phenolic profile and antioxidant activity [13, 14].

The many health advantages provided by Cornelian cherries recommend their use as functional ingredients in food and nutraceutical products. So, the extract must retain its characteristics and biological properties during storage. The stability of the bioactive components of the extracts is essential. The antioxidant activity of fruits extract can also be affected by storage conditions.

Many methods have been developed and tested in the literature; advantages and limitations of these methods have still been discussed, but there is no consensus regarding the most convenient assay as a standard method for claiming the total antioxidant capacity [15]. The most popular methods are based on ultraviolet-visible (UV-Vis) spectrometry, but chromatographic methods such as high-performance liquid chromatography (HPLC) or (high-performance) thin-layer chromatography (HP)TLC have also been used [16–18]. Several types of free radicals have been employed to estimate the scavenging capacity, but the ABTS^+∙^ decolorization assay has many advantages, namely, being more sensitive than the DPPH assay [19]; the reagent can be solubilized in both aqueous and organic media and is not affected by ionic strength, enables the measurement of both hydrophilic and lipophilic antioxidants, and so forth [15]. Moreover, ABTS assay was superior to the DPPH method in reflecting the antioxidant levels of foods containing hydrophilic, lipophilic, high moisture, or high pigmented nutrients so this method has been proved to be more useful in determination of the total antioxidant capacity of foods [20]. Antioxidant compounds also act as reductants. Their electron-donating capacity or reducing power is an important parameter in proving their antioxidant efficacy. This capacity can be determined through reactions with various metallic ions such as copper, iron, and cerium [21]. A typical electron-transfer based method is the ferric reducing antioxidant power (FRAP) assay, based on reduction of ferric ion (Fe^3+^) to Fe^2+^ and uses 2,4,6-tris-(2-pyridyl)-*s*-triazine (TPTZ) as ligand of the ferric ion. At low pH values, the reduction of this complex to the ferrous form results in an intense blue color which can be spectrophotometrically monitored by measuring the change in absorption at 593 nm [22].

Although the antioxidant capacity of Cornelian cherry fruits has been already investigated [13, 14, 23], there is no data about the variation of this parameter during storage at room temperature. The objective of this research was to study the variation of the antioxidant potential of Cornelian cherries extract using three different methods for the* in vitro* evaluation of the total antioxidant capacity, namely, high-performance thin-layer chromatography (HPTLC) and spectrophotometric ABTS and FRAP assays, in order to prove that the adding of Cornelian cherry fruits to human diet could increase the intake of exogenous antioxidants.

## 2. Materials and Methods

### 2.1. Plant Material and Reagents

Cornelian cherry fruits were purchased in August 2015 from a local market from Cluj-Napoca, Romania, washed with distilled water, and used directly for obtaining a concentrated extract.

The analytical grade chemicals and reagents, acetone, 2,2-azinobis(3-ethylbenzothiazolyne-6-sulphonic acid) diammonium salt (ABTS), 2,4,6-tris-(2-pyridyl)-*s*-triazine (TPTZ), potassium persulfate, 6-hydroxy-2,5,7,8-tetramethylchromane-2-carboxylic acid (Trolox), FeCl_3_,* n*-butanol, and formic acid, were purchased from Merck (Darmstadt, Germany). The silica gel 60F_254_ HPTLC plates, 10 × 10 cm, were also obtained from Merck (Darmstadt, Germany).

### 2.2. Preparation of Fruits Extract

Fresh Cornelian cherry fruits were milled after stone removal. Ten grams of milled fruits was mixed with 100 mL of acetone, stirred for 1 h at room temperature, and then vacuum-filtered. The acetone containing filtrate was concentrated under reduced pressure at 40°C on a rotary evaporator Buchi water bath, B480 (Buchi Labortechnik AG, Flawil, Switzerland) to 7.5 mL. The crude concentrated extract was stored for 19 days in the dark at room temperature. Samples were taken at different time intervals (0, 4, 7, 11, 16, and 19 days) and used to determine the antioxidant capacity as well as the variation of this parameter during storage.

### 2.3. Spectrophotometric Evaluation of Antioxidant Capacity

#### 2.3.1. ABTS Assay

The radical scavenging capacity of the extract was determined by the ABTS assay, according to the method described by Arnao et al. [24] with slight modifications [23]. The ABTS solution was prepared by dissolving 360 mg of ABTS in 100 mL distilled water. The activation of the ABTS^+∙^ was achieved by adding 100 mL ABTS solution in 100 mL of potassium persulfate solution 2.45 mM. The resulting mixture was kept in the dark for 24 h until the reaction was complete. The absorbance of the resulting solution measured at 734 nm was adjusted around 0.8 by dilution with distilled water. The 128-fold diluted fruit extract (0.1 mL) was added to 6 mL diluted ABTS solution and the mixture was kept in the dark for 15 minutes. The absorbance of the sample and blank was measured at 734 nm using a Perkin Elmer Lambda 25 double beam UV-Vis spectrophotometer (Perkin Elmer, Shelton, CT, USA). The antioxidant capacity of the investigated extract was expressed in *μ*mol Trolox equivalents/g fruits using a calibration curve (0–400 *μ*mol/L Trolox) obtained in the same conditions.

#### 2.3.2. FRAP Assay

The FRAP assay was conducted according to Benzie and Strain [22] with slight modifications [23]. The fresh working FRAP solution was prepared by adding 2.5 mL 0.01 M TPTZ (2,4,6-tripyridyl-*s*-triazine) solution in HCl and 2.5 mL 0.02 M FeCl_3_·6H_2_O solution to 25 mL 0.3 M acetate buffer (pH = 3.6). The resulting mixture was warmed at 37°C. 150 *μ*L fruit extract was mixed with 2850 *μ*L FRAP solution and incubated in the dark for 30 minutes. The absorbance of the resulted complex was measured at 593 nm against a blank sample. A standard curve (0–400 *μ*mol/L Trolox) was used to express the obtained absorbance values in *μ*mol Trolox equivalents.

### 2.4. HPTLC Evaluation of Antioxidant Capacity

Chromatographic evaluation of the antioxidant capacity was done after separation of polyphenols from the fruit extract on silica gel plates using as mobile phase a mixture of* n*-butanol-formic acid-water, 12 : 3 : 4.5 v/v/v. Samples of ten times diluted extract (5 *µ*L) were applied as 7 mm bands at 1.5 cm from the low edge of the plate with a rate of 30 nL/s using a semiautomatic applicator device (Linomat 5, Camag) at different time intervals (0, 4, 7, 11, 16, and 19 days). The compounds were detected under UV light (366 nm) and then the plate was immersed in ABTS^+∙^ solution, when the antioxidant compounds appeared as white zones on a green background. The plate was covered with aluminum foil and kept in the dark between each application of the extract. The images of the plate taken with Digistore 2, CAMAG, at 15 min after immersion was digitally processed in natural color using ImageJ computer software. The antioxidant capacity was determined on the basis of a calibration curve [19] and was expressed as *μ*mol Trolox/g fresh fruit.

### 2.5. Data Analysis

Data are reported as mean values of three experiments. Results were analyzed using one-way variance analysis (ANOVA). Analysis of variance was performed using XLSTAT Release 10 (Addinsoft, Paris, France). Differences at *p* < 0.05 were considered statistically significant.

## 3. Results and Discussion

The instability of natural extracts to temperature and long-time storage results in structural alterations that may affect their possible health benefits due to the changes of their antioxidant activity. The degradation pattern depends on both storage time and temperature. Processing or storage may sometimes improve the nutritional quality of some foods.

The variation of the antioxidant capacity of Cornelian cherries extract was determined using two* in vitro* antioxidant assays based on the ABTS method, which measures the capacity of antioxidants to perform as free radical scavengers and one typical electron-transfer based method (FRAP) in order to estimate the reducing power of the antioxidant compounds. Cornelian cherries are known to contain quercetin glycosides, anthocyanins, and kaempferol glycosides which are the major contributors to antioxidant capacity measured by a free radical scavenging assay [10, 25]. The antioxidant activity of fruits and vegetables depends in a great extent on the presence of the polyphenols and also on other compounds such as ascorbic acid. In order to isolate the antioxidant compounds and to identify the free radical scavenging capacity of each bioactive component in the fruit extract, the ABTS assay can be combined with prior HPTLC separation of these compounds, method which allows a better estimation of the total antioxidant capacity, compared to the widely used method of the spectrophotometric assessment of the antioxidant power of the crude extract. The screening of the antiradical power by thin-layer chromatography provides a rapid and easy way to investigate the plant extracts profile [26]. This method requires no sample purification and provides a simultaneous separation and determination of the radical scavenging capacity of antioxidant compounds present in the fruit extract. In order to provide a real profile of the total antioxidant capacity of the Cornelian cherry fruit extract, three different assays were applied, as one single method of estimating the antioxidant capacity is possible to not accurately reflect all antioxidants due to the complex composition of the sample [19].

Table 1 presents changes in the antioxidant capacity of the investigated extract during storage at room temperature (22°C).

Fluctuations in the antioxidant capacity with transient increases followed by decreases were noted when commonly used spectrophotometric ABTS and FRAP assays were applied. The decrease in the antioxidant capacity may be linked to losses of water soluble antioxidants such as phenolic compounds and vitamin C in the stored extracts and interactions with other compounds that alter the antioxidant capacity while the increase of the antioxidant property is usually assigned to Maillard reaction's products [27, 28]. ABTS assay indicated a 6.1% decrease of the antioxidant capacity of Cornelian cherry fruits extract after 19 days of storage at room temperature (22°C), while FRAP assay revealed a 3.2% decrease of this parameter.

**Figure 1:**
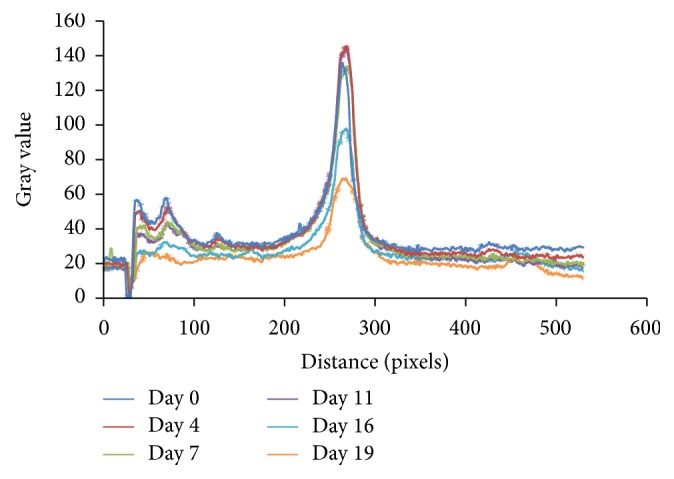
HPTLC chromatograms of Cornelian cherries extract stored at 22°C, obtained at 366 nm.

The experimental results (Table 1) show that the antioxidant capacity is approximately constant during storage varying between 12.91 and 12.83 *µ*mol Trolox/g FW. Thus, at the end of the storage period the antioxidant capacity was not significantly reduced, the decrease being only 0.62%. This behaviour apparently suggests that the composition of the extract does not change during storage. The assumption is contradicted by the HPTLC chromatograms (Figure 1). From this figure it can be seen that slight modification of the characteristic peaks occurs, while other new compounds appear, indicating a change in the composition of the fruit extract. These remarks lead us to conclude that the new compounds formed during storage possess comparable antioxidant capacity to that of the bioactive compounds which degrade.

The comparison of different methods used for antioxidant capacity determination is somewhat difficult due to the limitations in determination of hydrophilic antioxidants, the main problems occurring in establishing the reaction end point, light sensitivity of initiators or probes, possible interferences with other components, the use of different standards for expressing results, and so forth [15], although the comparison between the total antioxidant capacity determined by spectrophotometric and HPTLC methods reveals the synergistic action of antioxidant constituents of Cornelian cherries extract. This statement is proved by the lower antioxidant capacity values obtained by HPTLC bioassay. This behaviour is due to the fact that the global antioxidant capacity determined by this method is a sum of individual antioxidant activities of previously separated compounds. In the case of spectrophotometric ABTS and FRAP assays, the antioxidant capacity is determined for the whole mixture of all antioxidant compounds present in the investigated sample. It is well known that the mixture of antioxidant compounds could have a synergistic, antisynergistic, or additive effect [29]. The experimental results prove the synergistic effect of antioxidants from Cornelian cherries extract. Although the absolute values of the determined antioxidant capacity by the spectrophotometric methods differ from those determined by HPTLC, the variation of this parameter during storage followed the same trend in both cases.

## 4. Conclusions

The present study provided information on the variation of the antioxidant capacity of Cornelian cherries extract during storage at room temperature. The results suggest that an approximated 3 weeks of storage of the extract at room temperature does not alter the nutraceutical value and the health beneficial properties of these fruits and recommend them to be used as concentrated source of antioxidant compounds to develop functional foods with added health benefits. The comparative evaluation of antioxidant capacity by applying the three different methods proves to be a useful tool in investigation of synergetic/antisynergistic effect of antioxidant compounds mixtures.

## Figures and Tables

**Table 1:** Variations of antioxidant capacity of Cornelian cherries extract stored at 22°C.

Day	Total antioxidant capacity (*µ*mol Trolox/g FW)_ _^*∗*^		
	ABTS assay	FRAP assay	HPTLC method
0	36.13 ± 1.18_ _^a^	33.51 ± 1.27_ _^a^	12.91 ± 0.11_ _^a^
4	34.15 ± 1.43_ _^a^	31.56 ± 1.08_ _^a^	12.90 ± 0.15_ _^a^
7	35.27 ± 1.58_ _^a^	32.54 ± 1.39_ _^a^	12.88 ± 0.09_ _^a^
11	32.36 ± 1.34_ _^a^	30.97 ± 0.98_ _^a^	12.87 ± 0.04_ _^a^
16	36.82 ± 1.12_ _^a^	34.02 ± 1.25_ _^a^	12.85 ± 0.10_ _^a^
19	33.93 ± 1.09_ _^b^	32.43 ± 1.12_ _^b^	12.83 ± 0.07_ _^a^

__^*∗*^Data are given as mean value ± standard deviation. Different letters in each column indicate significant differences at 95% confidence level as obtained by LSD test.
